# Authors’ response to the Letter to the Editor by Efsun Somay and Erkan Topkan regarding our study “The influence of antibiotic administration on the outcomes of head-and-neck squamous cell carcinoma patients undergoing definitive (chemo)radiation”

**DOI:** 10.1007/s00405-023-07960-8

**Published:** 2023-04-13

**Authors:** Alexander Rühle, Jiadai Zou, Ilinca Popp, Nils H. Nicolay

**Affiliations:** 1 Department of Radiation Oncology, University of Freiburg-Medical Center, Robert-Koch-Str. 3, 79106 Freiburg, Germany; 2 German Cancer Consortium (DKTK), Partner Site Freiburg, German Cancer Research Center (DKFZ), Heidelberg, Germany; 3 Department of Radiation Oncology, University of Leipzig, Leipzig, Germany; 4 Comprehensive Cancer Center Central Germany, Partner Site Leipzig, Leipzig, Germany

We thank Dr. Somay and Dr. Topkan for the interest in our study about the association between antibiotic application and oncological outcomes in head-and-neck squamous cell carcinoma (HNSCC) patients undergoing (chemo)radiation [1]. We would like to shortly respond to their letter, in which they addressed the unequal distribution in the treatment type and cancer stage in our cohort, two aspects that are closely linked [2].

First, the number stated in the Letter to the Editor regarding the differences of concomitant systemic treatment is not correct: while 91.6% of patients in the antibiotic treatment group were treated with concomitant radiotherapy and systemic treatment (mostly chemotherapy), 57.6% of patients in the no-antibiotics group (and not 38.5%, as stated in the Letter to Editor) received concomitant systemic treatment. The difference is mainly related to the fact that the proportion of patients with early HNSCCs (UICC stages I–II) was higher in the no-antibiotics group than in the antibiotics group (22.7% versus 3.2%). It should be noted that systemic treatment was included in the multivariate Cox regression analysis of our study, so that we controlled for this imbalance. Furthermore, the vast majority of patients (about four out of five patients) received definitive chemoradiation in our analysis, so that results are largely driven by this treatment type. Given the complex interaction between antibiotic treatment, anti-cancer drugs and the microbiome in modulating the anti-cancer response (reviewed in Ref. [3]), we, however, agree with our colleagues that further analyses are required to clarify differences in the role of antibiotic treatment between radiotherapy, chemoradiation and cetuximab-based bioradiotherapy (and maybe in future, (chemo)radiation plus immune checkpoint inhibitors).

The second point that was raised concerned the unequal distribution of locoregionally advanced HNSCC patients between both groups, i.e., more patients in the antibiotic treatment group suffering from locoregionally advanced HNSCC. As we acknowledge this imbalance, we additionally conducted a subgroup analysis in which only patients with locoregionally advanced HNSCCs (UICC stages III–IV) were analyzed and also provided these data in the manuscript. In line with the results of the entire cohort, both overall survival and progression-free survival were significantly diminished in patients with locoregionally advanced HNSCCs that received antibiotics during the course of (chemo)radiation. There also was a non-significant trend towards reduced locoregional control in patients with locoregionally advanced HNSCCs exposed to antibiotics during (chemo)radiation (*p* = 0.084). It should be pointed out that a second analysis on this topic (in which the negative association between antibiotic treatment and treatment outcomes was also demonstrated) only incorporated patients with locoregionally advanced HNSCC [4]. In that multivariate regression analysis including other patient characteristics such as age, sex, stage, primary and nodal status, anticancer treatment and tumor localization, antibiotic treatment remained an independent prognosticator for impaired overall survival, progression-free survival and disease-specific survival. Univariate and multivariate Cox regression analyses (in which the primary and nodal status as well as the UICC stage were included) were also performed in our study, and at least in the subgroup of patients < 75 years, antibiotic treatment was an independent prognostic variable. Therefore, it is conceivable that the negative association between antibiotic treatment and impaired oncological outcomes is not solely related to differences in patients’ cancer stage. While we appreciate Dr. Somay’s and Dr. Topkan’s suggestion to balance both treatment groups by propensity score matching, we did not deem this approach feasible due to the limited sample size of 220 patients in our cohort and the number of variables (age, ECOG, tumor stage, nodal stage, UICC stage, tumor localization, concomitant systemic treatment) that would need to be incorporated.

In summary, the points raised by Dr. Somay and Dr. Topkan show the necessity for further prospective analyses including microbiome analyses to increase the understanding of the impact of peritherapeutic antibiotic treatment on treatment outcomes of HNSCC patients. Research efforts from our team as well as various other research groups are currently underway to further clarify the interaction of antibiotic treatment and HNSCC outcomes, and we are looking forward to additional data on this important topic.

## Data Availability

The data that support the findings of this study are available on reasonable request from the corresponding author.
